# Genome-wide DNA methylation profiling of CD4^+^ T lymphocytes identifies differentially methylated loci associated with adult primary refractory immune thrombocytopenia

**DOI:** 10.1186/s12920-023-01557-0

**Published:** 2023-06-08

**Authors:** Hanzhi Du, Qinghua Tang, Jian Yang, Bin Yan, Lihong Yang, Mengchang Wang

**Affiliations:** 1 Department of Haematology, the First Affiliated Hospital of Xi’an Jiaotong University, Xi’an, China; 2 Department of Osteoporosis, Xi’an Jiaotong University Affiliated HongHui Hospital, Xi’an, China; 3 Department of Clinical Research Centre, the First Affiliated Hospital of Xi’an Jiaotong University, Xi’an, China

**Keywords:** Primary refractory ITP, DNA methylation, CD4^+^ T lymphocytes

## Abstract

**Background:**

DNA methylation played a crucial role in the pathogenesis of immune thrombocytopenia (ITP). However, genome-wide DNA methylation analysis has not been applied thus far. The present study aimed to provide the first DNA methylation profiling for ITP.

**Methods:**

Peripheral blood CD4^+^ T lymphocytes samples were collected from 4 primary refractory ITP cases and 4 age-matched healthy controls, and DNA methylome profiling was performed using Infinium MethylationEPIC BeadChip. Differentially methylated CpG sites were further validated in another independent cohort of 10 ITP patients and 10 healthy controls using qRT-PCR.

**Results:**

The DNA methylome profiling identified a total of 260 differentially methylated CpG sites mapping to 72 hypermethylated and 64 hypomethylated genes. These genes were mainly enriched in the actin nucleation of the Arp2/3 complex, vesicle transport, histone H3-K36 demethylation, Th1 and Th2 cell differentiation, and Notch signaling pathway according to the GO and KEGG databases. The mRNA expression of CASP9, C1orf109, and AMD1 were significantly different.

**Conclusions:**

Given the altered DNA methylation profiling of ITP, our study provides new insights into its genetic mechanism and suggests candidate biomarkers for the diagnosis and treatment of ITP.

**Supplementary Information:**

The online version contains supplementary material available at 10.1186/s12920-023-01557-0.

## Introduction

Primary immune thrombocytopenia (ITP) is an autoimmune disease, primarily characterized by increased autoantibody-mediated platelets destruction and impaired platelets production. It was reported that primary ITP in adults affects 3.3 per 100,000 every year and it is more common in women and elderly patients [1]. Despite the variety and heterogeneity of the clinical presentations, decreased platelet counts and various bleeding symptoms are the most common manifestations [2]. A recurrence or progression to refractory disease with poor clinical results, markedly reduced quality of life, and elevated mortality affects about 30% of adult patients with primary ITP [3].

Although some studies have explored the pathogenesis of ITP, the underlying mechanisms are still incompletely elucidated. Immunological abnormalities are found to variable degrees in patients and their close relatives [4]. Immunosuppressive cells, primarily CD4^+^CD25^+^ regulatory T cell (Treg) subsets, have been shown to play an important role in the occurrence of ITP [5]. Treg cells are subsets of CD4^+^ T cells, and the proportion of these cells is crucial for maintaining immunological homeostasis, particularly when it comes to regulating cell differentiation and cellular immunity homeostasis. Serious autoimmune disorders frequently result from Treg cell malfunction. In autoimmune diseases like rheumatoid arthritis, systemic lupus erythematosus, and psoriasis, abnormal DNA methylation and Treg cell lineage dysfunction are common findings, and the blood DNA methylation of particular genes may serve as a biomarker for autoimmune diseases [6–9].

DNA methylation can maintain the stability of genetic material and regulate gene expression without altering nucleotide sequences. The loss of methylation homeostasis in immune cells may trigger the overexpression of methylation-related genes, hence contributing to the onset and progression of immunological thrombocytopenia [10, 11]. Previous studies have demonstrated that aberrant lymphocyte methylation is positively correlated with pathophysiology of ITP, presenting with a rising plasma SAH concentration and decreased expression levels of DNMT3A and 3B [12]. Studies have shown that the methylation-sensitive genes CD70, FOXP3, and IL-4 become involved in autoimmune reactions as a result of increased DNA methyltransferase (DNMTs) transferase activity that results from the chronic progression of ITP [13, 14]. In addition, decitabine and other low-dose demethylation medications may promote megakaryocyte maturation and platelet production [15]. Clinically, we also found that low-dose decitabine has improved the outcomes in patients with ITP [16]. These also suggest that DNA methylation modification is critical for the emergence and development of ITP. A potent approach for identifying methylation sites linked to illnesses is genome-wide DNA methylation profiling technology. Genome-wide DNA methylation profiling, however, has not yet been applied for ITP. The present study aims to provide a comprehensive assessment of DNA methylation differences in peripheral blood CD4^+^ T lymphocytes in primary refractory ITP.

## Materials and methods

### Study population

From June to December 2020, patients (age range:18–35) with refractory ITP, who attended the outpatient or inpatient departments in the First Affiliated Hospital of Xi’an JiaoTong University were incorporated into this study. Diagnosis and typing were assigned according to the Chinese guideline on the diagnosis and management of adult primary immune thrombocytopenia (version 2020) [17]. The 14 patients as subjects were randomly selected from the eligible patients and 14 age-matched healthy subjects were enrolled as the control group in this study. CD4^+^ T lymphocytes from 4 patients with primary refractory ITP and 4 healthy individuals were randomly collected for genome-wide DNA methylome profiling analysis and the differential methylation analysis between the two groups. The expression of particular genes in differential analysis was subsequently confirmed by qRT-PCR (Table 1). Ethnicity, age, gender, family history, and platelet count were collected from self-administered questionnaires and medical records. All of the study participants were Chinese Han individuals. Patients with secondary thrombocytopenia and a medication history of DNA-demethylating agents were excluded. The present study met with approval by the Medical Ethics Review Committee of the First Affiliated Hospital of Xi’an JiaoTong University (No: XJTU1AF2020LSK-021).

**Table 1:** Basic characteristics of the study subjects

Group	Refractory ITP			Healthy controls	
	Age(years)	Gender		Age(years)	Gender
Methylation chip					
1	29	Female		27	Male
2	28	Female		26	Male
3	27	Female		25	Male
4	26	Female		26	Female
qRT-PCR					
1	28	Female		27	Female
2	35	Male		30	Female
3	26	Male		25	Female
4	27	Female		26	Male
5	26	Male		26	Female
6	30	Male		30	Female
7	28	Female		27	Male
8	35	Female		34	Female
9	29	Male		28	Female
10	34	Male		31	Female

ITP: primary immune thrombocytopenia, qRT-PCR: quantitative real-time reverse transcription-polymerase chain reaction

### CD4^+^T lymphocytes sorting, purification and DNA extraction

The 10 ml of peripheral blood from each subject was obtained using EDTA-anticoagulant tubes. PBMCs were isolated by density gradient centrifugation using the Ficoll lymphocyte separation solution (Solarbio, China) from 10ml peripheral blood. CD4^+^ T lymphocytes were purified from PBMCs by positive selection using magnetic bead sorting (Miltenyi Biotec). Genomic DNA was extracted from the CD4^+^ T lymphocytes using the QIAamp DNA Blood Mini kit (Qiagen) following the manufacturer’s recommended instructions. Total DNA quantity and quality were checked by NanoDrop ND-2000 Spectrophotometer (Thermo Fisher Scientific Inc., USA). The concentration of each DNA sample was adjusted to 50ng/µL and the samples were then detected by 0.8% agarose gel electrophoresis in this study. The DNA sample with the purity (A260/280) varying from 1.8 to 2.0 was used in further experiments.

### Genome-wide DNA methylation profiling

The genome-wide DNA methylome assay was evaluated using Infinium MethylationEPIC BeadChip (Illumina, Inc.) according to the manufacturer’s protocol. Genomic DNA was bisulfite-converted with the Zymo EZ DNA Methylation kit (Zymo Research, USA). Bisulfite-converted DNA was hybridized to Infinium MethylationEPIC BeadChip, scanned with iScan system (Illumina, Inc), and processed using GenomeStudio software following the manufactures’ protocol. CpG probes with genetic variants and probes identified as cross-hybridizing were excluded. The methylation level of each CpG site was computed as a β value ranging from 0 to 1 (0 = not methylated, 1 = fully methylated). The BeadChip array covers 99% promoters, 95% CpG islands, enhancer regions and gene coding regions.

### Differential methylation analysis

The methylation data of 742,791 probes were obtained using the GenomeStudio software. The ChAMP package (2.28.0) was used for preprocessing, standardization, and quality control of the original data. Based on the top 1000 most variable probes in all samples, MDSplot (Supplementary Fig. 1) was used to visualize the similarity of samples. After original data filtration, quality control and normalization, differential methylation analysis was conducted with R package Limma (3.32.10). The correlations of samples were calculated following normalized β values. FDR-correction p-value was calculated by the Benjamini-Hochberg multiple hypothesis testing methods. The methylated CPG sites with an adjusted p＜0.05 and a |β difference|＞0.2 were considered significant. Differentially methylated regions were identified by the Bumphunter package (1.32.0).

### Functional enrichment analysis

To identify pathways and system biological processes of differentially methylated genes, GO terms and KEGG pathways were implemented by KOBAS (v3.0), taking all the methylation loci on the platform as the background. The significant differences were identified by KOBAS from the genome comparisons. P values were analyzed by χ^2^-test and corrected using Benjamini–Hochberg (BH) method. The genes assigned to encode transcripts or related biological processes were annotated based on GO. GO terms were divided into three categories: Molecular Function (MF), Biological Process (BP) and Cellular Component (CC). The KEGG database was used for metabolic processes and functional analysis.

### qRT-PCR validation

The transcriptional expression of a select panel of significantly differential methylated genes was analyzed by qRT-PCR. Total RNA was isolated from CD4^+^ T lymphocytes by RNA isolation solution (Sevicebio). The quality and concentration of isolated RNA were evaluated by an ND-2000 spectrophotometer (Thermo Scientific). Total RNA was converted into cDNA using a Servicebio®RT First Strand cDNA Synthesis Kit (Sevicebio). Subsequently, qRT-PCR was set up with 2×SYBR Green qPCR Master Mix (Sevicebio) following the manufacturers’ protocol. The primer sequences of each gene are shown in Additional File 1. In the analysis of qRT-PCR results, GAPDH was used as a housekeeping gene for normalization and the 2 −(ΔΔCT) analysis method was used for relative mRNA expression level. The *Student t-test* was used for normally distributed data and the *Mann-Whitney test* for non-normally distributed data. P value were adjusted using the Bonferroni correction method to determine the threshold of significance. Therefore, P = 0.007 (0.05/7) is used as the threshold of significant difference after multiple corrections.

## Results

### Differential DNA methylation analysis

**Fig. 1:**
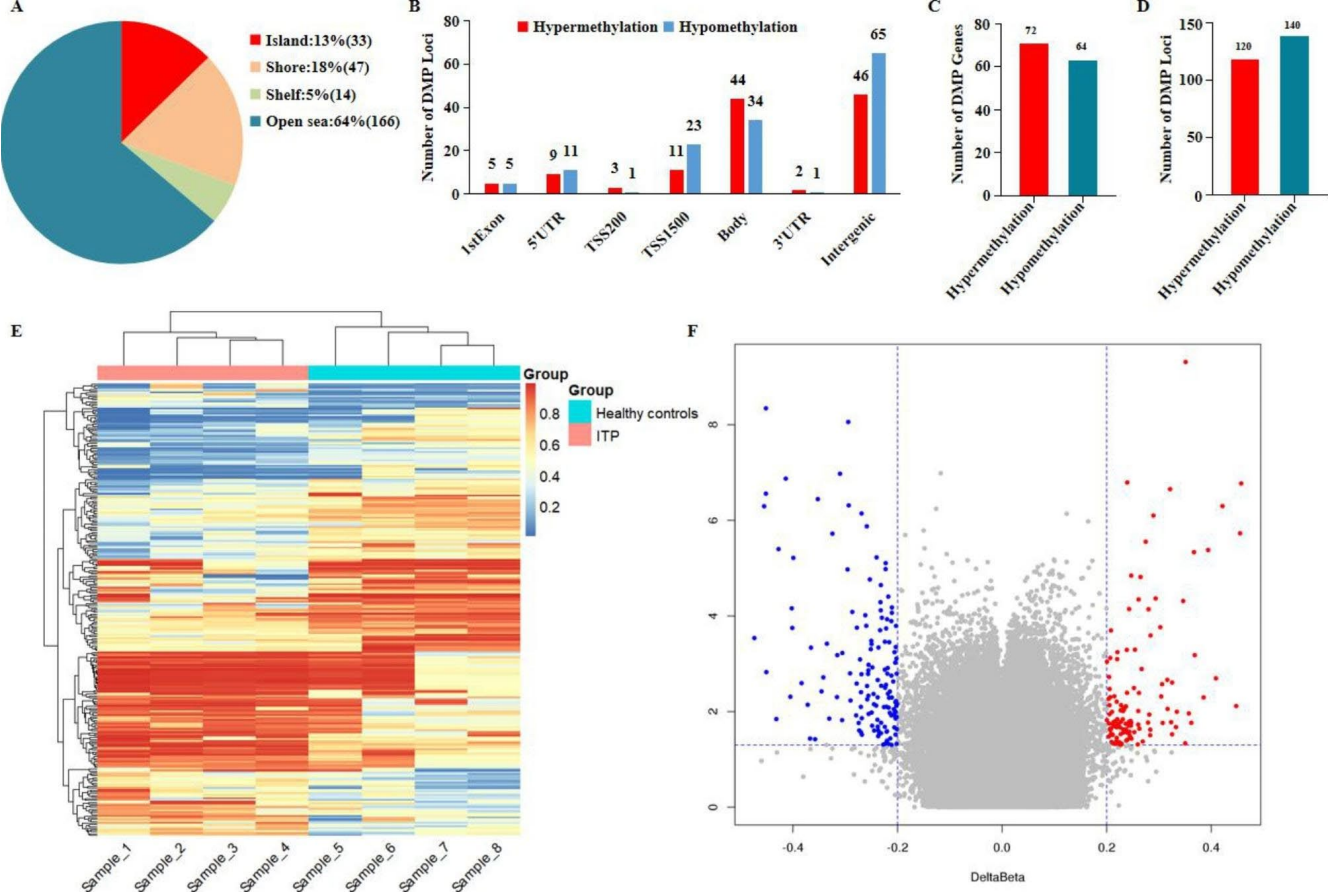
Characteristics of differential DNA methylation sites. **A**: Distributions of CPG sites (CPG island: CG-rich regions, CpG island shore: 2 kb regions flanking a CpG island, CpG shelf: 2 kb region outside a CpG shore, open sea: other regions besides CpG islands, CpG shores, and CpG shelves). **B**: Distribution of CPG in gene region. **C**: Number of differentially methylated genes. **D**: Number of differentially methylated loci. **E**: Heat map of differentially methylated CPG sites (Each column is a sample and each row represents methylation level, red and blue indicates higher and lower methylation, respectively. The top color bar reflects the grouping of samples, and there exists a significant difference in DNA methylation between the two groups). **F**: Volcano plot of the differentially methylated loci (abscissa scale is DeltaBeta, ordinate scale is –log10 (P values), red dots signify hypermethylated regions and blue hypomethylated, and grey regions designate loci with no significant difference)

A total of 742,791 CPG loci were detected and 19% of them were distributed in CPG islands (Supplementary Fig. 2A). Using the screening conditions of P < 0.05 and ∣△β∣>0.20, we found 260 CPG sites to be significantly methylated. About 64% of these sites were located in the open sea region and others were in the gene promoter regions and adjacent upstream and downstream regions (Fig. 1A). As shown in Fig. 1B, most of the differentially methylated loci are distributed in the intergenic region, followed by the body region, and about 1/3 of them are located in the promoter region (TSS1500, TSS200, 5’UTR and 1st Exon). Supplementary Fig. 2B displays the distribution of the differential methylated CPG sites in the genome. Across all differentially methylated sites, the hypermethylated sites accounted for 46.2%, including 120 sites corresponding to 72 genes, while the hypomethylated sites accounted for 53.8%, including 140 sites corresponding to 64 genes (Fig. 1C and D). We conducted a clustering analysis based on the methylated CpG sites and their methylation levels, the cluster diagram of differential CpG revealed that there exhibits differential methylation between the two groups (Fig. 1E). We also displayed the volcano plot that can visually reflect the number of differential loci and significant differences between the two groups. The closer the points are to the top left corner and the top right corner of the map, the more significant the differences (Fig. 1F). Differential hyper- and hypo-methylated sites were summarized according to the differentially methylated genes screening (Additional File 2; Additional File 3) and performed functional enrichment analysis. The identified significant differentially methylated loci are shown in Table 2. We additionally identified six differentially methylated regions (DMRs). Of these, one DMR is up-regulated and the other five DMRs are down-regulated. Most of the DMPs covered by them are also located in the promoter region (exon 1, 5’UTR and TSS) and they are mainly located in CPG island (Additional File 4).

**Table 2:** List of top ten hypermethylated loci and top eight hypomethylated loci

Index	probeset	*P* Value	DeltaBeta	Gene Name
Hypermethylation	cg05513408	1.87E-06	0.455	KRTAP3-1
	cg04143156	1.43E-05	0.247	ARFIP1
	cg01879420	4.87E-05	0.346	AMD1
	cg18379824	7.16E-05	0.243	CASP9
	cg12322605	1.71E-04	0.303	CYFIP1
	cg06917450	5.07E-04	0.254	C1orf109
	cg04663285	5.72E-04	0.221	B4GALNT4
	cg08751451	6.61E-04	0.369	PRKG1
	cg19980771	7.57E-04	0.207	SLC22A16
	cg13217931	8.00E-04	0.22	ZNF148
Hypomethylation	cg05213296	8.73E-09	-0.294	RPF2
	cg08477332	1.06E-07	-0.31	S100A14
	cg02050512	1.34E-07	-0.414	AMPH
	cg24432675	2.76E-07	-0.452	ADARB2
	cg15718932	7.86E-06	-0.223	GBP2
	cg06952129	1.04E-05	-0.223	RASGRF2
	cg17776284	8.60E-05	-0.211	S100A13
	cg26446133	9.66E-05	-0.262	CNDP2

### GO and KEGG pathway functional enrichment analysis

To investigate the features of these differentially methylated genes, GO functional annotation and KEGG enrichment analysis were performed. As illustrated in Additional File 5, we found that these genes were highly riched in GO terms including regulation of Arp2/3 complex-mediated actin nucleation (P = 2.90 × 10^− 4^), histone H3-K36 demethylation (P = 1.86 × 10^− 3^), receptor recycling (1.43 × 10^− 3^), toll-like receptor 4 signaling pathway (1.68 × 10^− 3^), synaptic vesicle recycling (P = 1.91 × 10^− 3^). As shown in Tables 3 and 12 significantly differential KEGG enrichment pathways were identified for differentially methylated genes, such as Th1 and Th2 cell differentiation, Notch signaling pathway and PI3K-Akt signaling pathway.

**Table 3:** KEGG pathway enrichment analysis of differentially methylated genes between ITP patients and healthy controls

KEGG ID	Description	DMGs	*P* value
hsa04658	Th1 and Th2 cell differentiation	4	1.39E-03
hsa00330	Arginine and proline metabolism	3	2.40E-03
hsa05145	Toxoplasmosis	4	2.91E-03
hsa00250	Alanine, aspartate and glutamate metabolism	2	1.62E-02
hsa04975	Fat digestion and absorption	2	1.78E-02
hsa04919	Thyroid hormone signaling pathway	3	2.48E-02
hsa00270	Cysteine and methionine metabolism	2	2.51E-02
hsa04330	Notch signaling pathway	2	2.71E-02
hsa04270	Vascular smooth muscle contraction	3	3.21E-02
hsa00750	@@Vitamin B6 metabolism	1	3.52E-02
hsa04151	PI3K-Akt signaling pathway	5	3.63E-02
hsa05416	Viral myocarditis	2	3.67E-02

DMGs: Differential methylation genes

### qRT-PCR validation

**Fig. 2:**
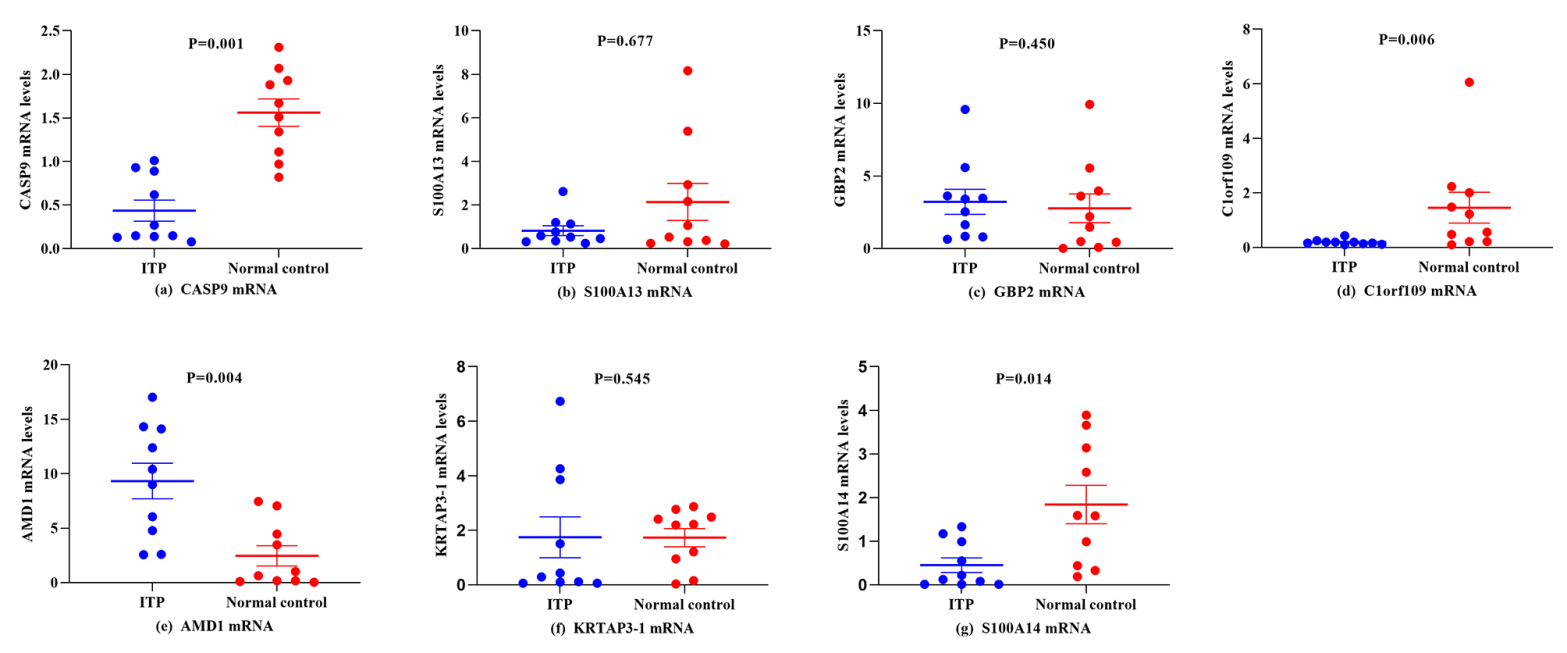
qRT-PCR results for CASP9 **(a)**, S100A13 **(b)**, GBP2 **(c)**, C1orf109 **(d)**, AMD1 **(e**), KRTAP3-1 **(f)**, S100A14 **(g)** mRNA expressions in CD4^+^ T lymphocytes from patients with ITP and healthy controls. The mRNA expression levels of CASP9 and C1orf109 in CD4^+^ T lymphocytes of ITP were significantly lower than that of control specimens (p＜0.007). The mRNA expression level of AMD1 in ITP group was higher than that in healthy control group (P = 0.004)

To further evaluate the biological function of identified differentially methylated genes, we screened out seven significant differentially genes (CASP9, S100A13, GBP2, Clor109, AMD1, KRTAP3-1, S100A14) from the top 10 hypermethylated and the top 8 hypomethylated sites according to the differential analysis results and the fact that they are located in the promoter region. At the same time, they were screened by combining relevant literature reports and referring to GO and KEGG enrichment analysis results. These genes were selected for qRT-PCR experiment to explore their mRNA expression level in CD4^+^ T lymphocytes between 10 ITP patients and 10 healthy control subjects. The mRNA expression levels of CASP9 and C1orf109 in CD4^+^ T lymphocytes of ITP were significantly lower than that of control specimens (p＜0.007). However, the mRNA expression level of AMD1 in the ITP group was higher than that in the healthy control group (P = 0.004). Compared with the healthy control group, there was no significant difference in the other four differential genes (P > 0.007). (Fig. 2)

## Discussion

Recent research has demonstrated how crucial DNA methylation is to the pathophysiology of primary ITP. We have found that demethylating drugs are beneficial for ITP patients in clinical treatment. Clarifying the role of DNA methylation in ITP may shed light on its pathogenesis and potential treatments.

The genome-wide DNA methylome profiling and CD4^+^ T cell DNA methylation differences in patients with primary refractory ITP were described in the current study. Functional enrichment analysis showed that differently methylated genes are mostly involved in platelet activation, platelet apoptosis homeostasis, platelet signal transduction, and DNA methylation regulation. It has been established that the DNA methylation modification contributes to the development of ITP. Then, the mRNA expression levels of candidate genes with differential methylation (CASP9, S100A13, GBP2, Clorf109, AMD1, KRTAP3-1, S100A14) were analyzed by qRT-PCR in an independent sample. Our results found that mRNA expression levels in CASP9, C1orf109 and AMD1 were different from those in healthy controls. Accordingly, it was hypothesized that CASP9, C1orf109, and AMD1 might be harnessed as therapeutic targets and provide a rationale for early diagnosis and treatment in patients with primary refractory ITP.

Recent studies have shown that altered activity or expression of CASP9 was involved in a variety of malignancies and autoimmune diseases [18]. We found that the mRNA expression of CASP9 is related to its hypermethylation in the promoter region. The mRNA expression of CASP9 in the ITP group was lower than the control group, resulting in apoptosis inhibition and imbalance of CD4^+^ T cell subsets and activating the cellular immune response. C1orf109 is a phosphorus-containing intracellular signaling protein molecule that may enter the nucleus to regulate gene expression. C1orf109 has never been implicated in ITP. Similarly, we found that the low expression of C1orf109 was associated with its hypermethylation in ITP and speculated that the level affects the proliferation and activation of CD4^+^ T lymphocytes and thus cellular immune response. AMD1 is involved in the decarboxylation of SAM and mainly affects the biological processes of DNA methylation and demethylation [19]. Additionally, AMD1 is a key enzyme in polyamine synthesis, mainly involved in the synthesis of spermine and spermidine. It has been shown that blocking polyamine reduces tumor invasion, apoptosis, angiogenesis, and cell proliferation. In this study, compared with the normal control group, we found that the expression of AMD1 was much higher. It is conceivable that AMD1 primarily affects megakaryocytes differentiation, which in turn has an impact on platelet production.

GO and KEGG analysis indicated significantly differential methylation genes were mainly enriched in immune-related pathways and the process of platelet production and activation such as regulation of actin nucleation mediated by Arp2/3 complex, Th1 and Th2 cell differentiation, Notch signaling pathway, vesicle transport process and histone H3-K36 demethylation. Arp2/3 complex has been shown to regulate actin assembly, which is involved in the formation of myofilament. AI Absi A et al. found that actin in breast cancer cells was concentrated in the immune synapses of tumor cells which decreased NK cell cytotoxicity. Actin altered the cytotoxic function of immune cells by driving the local recruitment of immunosuppressive ligands, suggesting that actin-mediated immune-escape mechanisms may be involved in the autoimmune process of ITP [20]. MAGEE C N found that compared with normal T cells, the proportion of Notch-1 in regulatory T cells increased after transplantation of patients’ blood, and the Notch signaling pathway has been proven to influence Tregs expansion and regulate the differentiation and function of T cells [21]. Microvesicles released by platelets have contributed to the differentiation from hematopoietic stem cells to megakaryocytes, and then promote the generation and activation of platelet [22]. TET2 and histone H3-K36 that recruited DNMT3B promote DNA demethylation and methylation, respectively [23]. Dynamic mutual regulation is important in the expression of coding genes contributing to the development of ITP.

The present study had several strengths and limitations. This is, to our knowledge, the first study focusing on the roles of genome-wide DNA methylation in CD4^+^ T lymphocytes from individuals with primary refractory ITP. Our results suggest the vital role of DNA methylation in the development of ITP and provide novel clues for elucidating the pathogenesis and therapeutics of primary refractory ITP. However, there were some limitations in this study. First, due to the tissue specificity of gene expression, the findings obtained from CD4^+^ T lymphocytes may not be applicable to other related cells or tissues. Further additional studies are warranted to corroborate our findings. In addition, limitations also include the lack of strict gender-matched and correction for confounding factors such as age and gender between the two groups. Future studies matching the gender, and correcting the confounding factors between groups and large samples will be required to clarify possible differences and their implications. Moreover, epigenetic data with genome-wide DNA methylation profiling and transcriptomics should be integrated to analyze the gene regulatory networks in the future.

## Conclusion

In conclusion, we performed genome-wide DNA methylation profiling of primary refractory ITP and healthy controls, and identified a set of differentially methylated genes and pathways. The present study contributes new insights into the genetic mechanism and provides candidate biomarkers for diagnosis and treatment of ITP.

## Electronic supplementary material

Below is the link to the electronic supplementary material.

Supplementary Material 1

Supplementary Material 2

Supplementary Material 3

Supplementary Material 4

Supplementary Material 5

Supplementary Material 6

Supplementary Material 7

Supplementary Material 8

Supplementary Material 9

Supplementary Material 10

Supplementary Material 11

## Data Availability

The datasets generated and analyzed during the current study are available in the Gene Expression Omnibus (GEO) database at https://www.ncbi.nlm.nih.gov/geo/query/acc.cgi?acc=GSE205495 (accession number: GSE205495).

## Abbreviations

ITP: immune thrombocytopenia
GO: Gene Ontology
KEGG: Kyoto Encyclopedia of Genes and Genomes
qRT-PCR: quantitative real-time reverse transcription-polymerase chain reaction PBMCs:peripheral blood mononuclear cells
