# Supplementary material for: Genome-wide DNA methylation profiling of CD4+ T lymphocytes identifies differentially methylated loci associated with adult primary refractory immune thrombocytopenia
Source: BMC Med Genomics. 2023 Jun 8;16:124. doi: 10.1186/s12920-023-01557-0 (PMC10251572; doi:10.1186/s12920-023-01557-0)
Supplement: Supplementary file 3 — Supplementary Material 3 [file 12920_2023_1557_MOESM3_ESM.docx]

**Additional file 2. List of differential methylation loci**

| **Index** | **probeset** | ***P* Value** | **DeltaBeta** | **Gene Name** |
| --- | --- | --- | --- | --- |
| Hypermethylation | cg27586797 | 4.87E-10 | 3.51E-01 |  |
|  | cg18697803 | 1.61E-07 | 2.39E-01 | SCN10A; SCN10A; SCN10A |
|  | cg20070837 | 1.69E-07 | 4.57E-01 | C14orf79 |
|  | cg04615850 | 2.22E-07 | 3.21E-01 | KDM4B |
|  | cg19070118 | 5.03E-07 | 4.21E-01 |  |
|  | cg23719516 | 7.95E-07 | 2.89E-01 |  |
|  | cg05513408 | 1.87E-06 | 4.55E-01 | KRTAP3-1 |
|  | cg27289463 | 2.80E-06 | 2.75E-01 |  |
|  | cg16342049 | 4.18E-06 | 3.94E-01 |  |
|  | cg24693760 | 4.63E-06 | 3.67E-01 |  |
|  | cg04143156 | 1.43E-05 | 2.47E-01 | ARFIP1; ARFIP1; ARFIP1;ARFIP1;ARFIP1;ARFIP1 |
|  | cg24933364 | 1.53E-05 | 2.65E-01 |  |
|  | cg11013060 | 4.30E-05 | 2.94E-01 |  |
|  | cg16217445 | 4.51E-05 | 2.61E-01 |  |
|  | cg01879420 | 4.87E-05 | 3.46E-01 | AMD1; AMD1; AMD1; AMD1; AMD1 |
|  | cg18379824 | 7.16E-05 | 2.43E-01 | CASP9; CASP9; CASP9; CASP9; CASP9 |
|  | cg00037728 | 7.22E-05 | 2.80E-01 | FAM208B |
|  | cg12322605 | 1.71E-04 | 3.03E-01 | CYFIP1; CYFIP1 |
|  | cg17715141 | 2.01E-04 | 2.08E-01 |  |
|  | cg00079566 | 2.55E-04 | 2.84E-01 | LINC00578 |
|  | cg06917450 | 5.07E-04 | 2.54E-01 | C1orf109 |
|  | cg07333763 | 5.13E-04 | 2.38E-01 |  |
|  | cg04663285 | 5.72E-04 | 2.21E-01 | B4GALNT4 |
|  | cg08751451 | 6.61E-04 | 3.69E-01 | PRKG1; PRKG1 |
|  | cg19980771 | 7.57E-04 | 2.07E-01 | SLC22A16 |
|  | cg13217931 | 8.00E-04 | 2.20E-01 | ZNF148 |
|  | cg13762612 | 9.06E-04 | 2.01E-01 |  |
|  | cg18434912 | 1.29E-03 | 2.67E-01 | AMD1; AMD1; AMD1; AMD1; AMD1 |
|  | cg19035788 | 1.89E-03 | 2.05E-01 |  |
|  | cg24088508 | 2.02E-03 | 4.09E-01 | C1orf109 |
|  | cg14706331 | 2.18E-03 | 3.16E-01 | MTTP; MTTP |
|  | cg14128857 | 2.47E-03 | 3.25E-01 |  |
|  | cg18902440 | 2.67E-03 | 3.06E-01 | HLA-DQB1 |
|  | cg21443480 | 3.21E-03 | 2.52E-01 | LOC284825 |
|  | cg27413643 | 3.37E-03 | 2.06E-01 | ANKRD27; RGS9BP |
|  | cg25117092 | 4.06E-03 | 2.39E-01 | MED12L; P2RY14; P2RY14 |
|  | cg25597117 | 4.08E-03 | 2.62E-01 |  |
|  | cg18865685 | 4.78E-03 | 2.15E-01 | ASTN1; ASTN1 |
|  | cg04154653 | 4.83E-03 | 3.04E-01 | TTLL10 |
|  | cg11839682 | 4.99E-03 | 2.07E-01 |  |
|  | cg07599136 | 5.06E-03 | 3.85E-01 | AHRR |
|  | cg07993845 | 5.41E-03 | 2.04E-01 |  |
|  | cg05593887 | 6.20E-03 | 2.19E-01 | MAGI2 |
|  | cg26664137 | 7.35E-03 | 2.28E-01 |  |
|  | cg21429687 | 7.39E-03 | 2.06E-01 | ANK3 |
|  | cg19976037 | 7.67E-03 | 4.48E-01 | CCDC57 |
|  | cg13046221 | 7.74E-03 | 2.37E-01 |  |
|  | cg02270332 | 7.88E-03 | 2.37E-01 |  |
|  | cg25079981 | 8.51E-03 | 2.33E-01 |  |
|  | cg03834478 | 8.80E-03 | 3.17E-01 |  |
|  | cg18562299 | 9.13E-03 | 2.33E-01 |  |
|  | cg11406134 | 9.41E-03 | 2.27E-01 | ALDH4A1; ALDH4A1; ALDH4A1 |
|  | cg13210558 | 9.73E-03 | 2.60E-01 |  |
|  | cg22089620 | 9.77E-03 | 2.31E-01 | PRM1 |
|  | cg09516963 | 1.01E-02 | 3.34E-01 | DYRK2; DYRK2 |
|  | cg02954704 | 1.02E-02 | 2.10E-01 |  |
|  | cg03393625 | 1.09E-02 | 3.57E-01 |  |
|  | cg22530767 | 1.14E-02 | 2.05E-01 | PRKCH |
|  | cg13503915 | 1.16E-02 | 2.82E-01 | SIGLEC5 |
|  | cg26710839 | 1.45E-02 | 2.20E-01 |  |
|  | cg11173447 | 1.45E-02 | 2.31E-01 |  |
|  | cg13159023 | 1.51E-02 | 2.00E-01 | PDXK |
|  | cg00102615 | 1.57E-02 | 2.46E-01 | NOTCH1 |
|  | cg15051063 | 1.59E-02 | 2.16E-01 |  |
|  | cg15487187 | 1.63E-02 | 2.43E-01 |  |
|  | cg11370814 | 1.65E-02 | 2.27E-01 |  |
|  | cg05354572 | 1.66E-02 | 2.13E-01 |  |
|  | cg03768569 | 1.67E-02 | 3.23E-01 |  |
|  | cg02099390 | 1.70E-02 | 2.79E-01 | OSBPL10 |
|  | cg13753488 | 1.71E-02 | 2.47E-01 | RALGDS |
|  | cg20701799 | 1.72E-02 | 3.62E-01 |  |
|  | cg09312897 | 1.73E-02 | 3.07E-01 | HLA-DQB1 |
|  | cg23680523 | 1.78E-02 | 2.05E-01 | TPCN1; TPCN1; TPCN1 |
|  | cg01380346 | 1.80E-02 | 2.36E-01 | ELL |
|  | cg12890080 | 1.83E-02 | 2.22E-01 |  |
|  | cg22763134 | 1.87E-02 | 2.60E-01 | MAML3 |
|  | cg27454650 | 1.89E-02 | 2.12E-01 | ANKS1A |
|  | cg27354323 | 1.93E-02 | 2.21E-01 | TENM3 |
|  | cg17656213 | 1.97E-02 | 2.46E-01 | PNMAL2; PPP5D1 |
|  | cg11769851 | 1.98E-02 | 2.14E-01 | KIAA1688 |
|  | cg20245116 | 1.99E-02 | 2.42E-01 | PCSK9 |
|  | cg10451773 | 2.10E-02 | 2.17E-01 |  |
|  | cg09645818 | 2.11E-02 | 3.33E-01 |  |
|  | cg06174908 | 2.13E-02 | 2.20E-01 | LRRN2; LRRN2 |
|  | cg24038762 | 2.16E-02 | 2.26E-01 |  |
|  | cg07413658 | 2.26E-02 | 2.08E-01 |  |
|  | cg18105134 | 2.28E-02 | 2.63E-01 | PROZ |
|  | cg27195001 | 2.31E-02 | 2.06E-01 |  |
|  | cg25744433 | 2.37E-02 | 2.37E-01 | CDH13; CDH13; CDH13; CDH13 |
|  | cg15612221 | 2.38E-02 | 2.32E-01 |  |
|  | cg02405213 | 2.39E-02 | 2.83E-01 | JAK2 |
|  | cg27366766 | 2.44E-02 | 2.07E-01 | HSF5 |
|  | cg08260054 | 2.64E-02 | 2.44E-01 | MAN2B2; MAN2B2 |
|  | cg00609466 | 2.70E-02 | 2.53E-01 |  |
|  | cg18970592 | 2.74E-02 | 2.32E-01 | OBSCN; OBSCN; OBSCN |
|  | cg24224180 | 2.75E-02 | 2.39E-01 | MINA; MINA; MINA; MINA; MINA; MINA; MINA;  MINA |
|  | cg23669081 | 2.97E-02 | 2.27E-01 | HOXB7 |
|  | cg18265608 | 2.98E-02 | 2.08E-01 | ACCS; ACCS |
|  | cg20947514 | 2.99E-02 | 2.11E-01 |  |
|  | cg11079896 | 2.99E-02 | 3.25E-01 | RHOJ; RHOJ |
|  | cg23246911 | 3.09E-02 | 2.11E-01 | RPH3AL |
|  | cg12782676 | 3.12E-02 | 2.83E-01 |  |
|  | cg02484732 | 3.23E-02 | 2.44E-01 | COL21A1 |
|  | cg09171253 | 3.33E-02 | 2.04E-01 | HPCAL1; HPCAL1 |
|  | cg22285475 | 3.57E-02 | 2.19E-01 | LINC01505 |
|  | cg00387872 | 3.68E-02 | 2.47E-01 |  |
|  | cg06113708 | 3.88E-02 | 2.40E-01 | COMTD1 |
|  | cg15882603 | 4.05E-02 | 2.37E-01 |  |
|  | cg02150060 | 4.22E-02 | 2.18E-01 | GLT6D1; GLT6D1 |
|  | cg03615426 | 4.23E-02 | 2.69E-01 | IQCK; IQCK; IQCK; IQCK; IQCK |
|  | cg14076451 | 4.32E-02 | 2.09E-01 | LOC154449 |
|  | cg18070442 | 4.39E-02 | 2.24E-01 | CACNA1I; CACNA1I |
|  | cg03173748 | 4.53E-02 | 2.26E-01 | CDH13; CDH13; CDH13; CDH13 |
|  | cg07189587 | 4.61E-02 | 3.50E-01 | RHOJ; RHOJ |
|  | cg16585183 | 4.71E-02 | 2.28E-01 | PRKG1; PRKG1 |
|  | cg17951884 | 4.72E-02 | 2.22E-01 | SGK2; SGK2; SGK2 |
|  | cg17086398 | 4.73E-02 | 2.15E-01 | SERINC2 |
|  | cg24247231 | 4.85E-02 | 2.28E-01 | MAP2K5; MAP2K5 |
|  | cg18786623 | 4.87E-02 | 2.59E-01 | HCG4P6 |
|  | cg07107105 | 4.95E-02 | 2.27E-01 | SCIMP; SCIMP; LOC100130950 |
| Hypomethylation | cg16224321 | 4.53E-09 | -4.52E-01 |  |
|  | cg05213296 | 8.73E-09 | -2.94E-01 | RPF2 |
|  | cg08477332 | 1.06E-07 | -3.10E-01 | S100A14 |
|  | cg02050512 | 1.34E-07 | -4.14E-01 | AMPH; AMPH |
|  | cg24432675 | 2.76E-07 | -4.52E-01 | ADARB2 |
|  | cg04419557 | 3.60E-07 | -3.52E-01 | RPF2; RPF2 |
|  | cg25059408 | 4.86E-07 | -2.93E-01 |  |
|  | cg03329597 | 5.07E-07 | -4.55E-01 | MYH15 |
|  | cg19500155 | 7.17E-07 | -2.69E-01 | TBCD |
|  | cg05510714 | 1.33E-06 | -2.59E-01 | KYNU; KYNU; KYNU |
|  | cg07450693 | 1.90E-06 | -3.25E-01 | MGMT |
|  | cg05890377 | 3.99E-06 | -4.28E-01 |  |
|  | cg18595228 | 5.95E-06 | -2.40E-01 |  |
|  | cg20891558 | 6.09E-06 | -3.99E-01 |  |
|  | cg15718932 | 7.86E-06 | -2.23E-01 | GBP2 |
|  | cg06952129 | 1.04E-05 | -2.23E-01 | RASGRF2 |
|  | cg16814680 | 1.07E-05 | -2.96E-01 |  |
|  | cg19729930 | 1.72E-05 | -2.53E-01 |  |
|  | cg12950816 | 2.26E-05 | -2.32E-01 | ZFAND2A |
|  | cg06086634 | 3.94E-05 | -2.18E-01 | MYCBPAP |
|  | cg03168497 | 5.15E-05 | -2.33E-01 | MYCBPAP |
|  | cg01345136 | 6.64E-05 | -2.11E-01 | GALNT9 |
|  | cg05779406 | 6.92E-05 | -4.02E-01 | ZFAND2A |
|  | cg27449200 | 7.63E-05 | -2.32E-01 |  |
|  | cg23785275 | 8.24E-05 | -2.86E-01 | HLA-DQB2 |
|  | cg17776284 | 8.60E-05 | -2.11E-01 | S100A13; S100A13; S100A13; S100A13; S100A13;  S100A13; S100A1 |
|  | cg26446133 | 9.66E-05 | -2.62E-01 | CNDP2; CNDP2; CNDP2 |
|  | cg06760111 | 1.09E-04 | -2.30E-01 | ZNF385D |
|  | cg14882966 | 1.17E-04 | -2.20E-01 |  |
|  | cg17592940 | 1.28E-04 | -2.12E-01 |  |
|  | cg25283121 | 1.61E-04 | -2.60E-01 | GALNT9 |
|  | cg03316587 | 1.61E-04 | -2.03E-01 |  |
|  | cg01786048 | 1.76E-04 | -2.78E-01 | ESPNL |
|  | cg25374269 | 1.78E-04 | -4.01E-01 |  |
|  | cg01017244 | 1.82E-04 | -2.22E-01 |  |
|  | cg22243260 | 1.99E-04 | -2.33E-01 |  |
|  | cg17212350 | 2.90E-04 | -4.74E-01 | ABCB4; ABCB4; ABCB4 |
|  | cg00048370 | 3.31E-04 | -2.50E-01 |  |
|  | cg01748496 | 3.47E-04 | -2.24E-01 | GLIS3; GLIS3 |
|  | cg10545854 | 3.54E-04 | -2.16E-01 | SPOP; SPOP; SPOP; SPOP; SPOP; SPOP |
|  | cg25291037 | 3.81E-04 | -3.35E-01 |  |
|  | cg20370296 | 3.87E-04 | -2.50E-01 | ARHGAP39 |
|  | cg12853970 | 4.47E-04 | -2.03E-01 |  |
|  | cg01601518 | 4.57E-04 | -2.36E-01 | ZFYVE28 |
|  | cg24593918 | 4.60E-04 | -3.66E-01 | HLA-DQB1 |
|  | cg23915527 | 4.95E-04 | -2.51E-01 |  |
|  | cg11161769 | 5.73E-04 | -2.06E-01 |  |
|  | cg17190362 | 5.95E-04 | -3.06E-01 | LAMA4; LAMA4; LAMA4 |
|  | cg09768983 | 6.61E-04 | -3.15E-01 |  |
|  | cg07030646 | 7.80E-04 | -2.02E-01 |  |
|  | cg09406933 | 8.12E-04 | -2.72E-01 | CTRB1 |
|  | cg00769632 | 9.49E-04 | -2.04E-01 | CENPU; CENPU |
|  | cg17298326 | 1.05E-03 | -2.56E-01 |  |
|  | cg06388363 | 1.19E-03 | -2.48E-01 |  |
|  | cg10249074 | 1.23E-03 | -2.19E-01 | C4orf11; C4orf11 |
|  | cg15007959 | 1.33E-03 | -2.24E-01 | SPIB |
|  | cg26872792 | 1.38E-03 | -2.56E-01 | PLA2G1B |
|  | cg21787089 | 1.49E-03 | -4.51E-01 | LTF; LTF |
|  | cg18561199 | 1.59E-03 | -2.91E-01 | SERPINA4; SERPINA4; SERPINA4 |
|  | cg11440486 | 1.61E-03 | -2.19E-01 | MYCBPAP |
|  | cg02174092 | 1.61E-03 | -2.02E-01 |  |
|  | cg08799779 | 1.63E-03 | -2.56E-01 |  |
|  | cg01166985 | 1.66E-03 | -2.69E-01 | RPH3AL; RPH3AL; RPH3AL; RPH3AL |
|  | cg03516004 | 1.77E-03 | -2.12E-01 | CTDP1; CTDP1; CTDP1 |
|  | cg19637330 | 1.93E-03 | -3.42E-01 |  |
|  | cg14851700 | 2.19E-03 | -2.38E-01 | GLUL; GLUL |
|  | cg10055637 | 2.34E-03 | -2.08E-01 |  |
|  | cg01427108 | 2.54E-03 | -3.84E-01 | LTF; LTF |
|  | cg03950493 | 2.59E-03 | -2.78E-01 | KIRREL3; KIRREL3 |
|  | cg22026953 | 2.76E-03 | -2.22E-01 |  |
|  | cg12468774 | 2.89E-03 | -2.31E-01 | CCDC36; CCDC36 |
|  | cg14029686 | 2.92E-03 | -2.58E-01 |  |
|  | cg01675596 | 3.17E-03 | -2.24E-01 |  |
|  | cg09976142 | 3.78E-03 | -3.45E-01 | CIZ1; CIZ1 |
|  | cg04344111 | 3.80E-03 | -2.15E-01 |  |
|  | cg00901687 | 3.94E-03 | -2.70E-01 | MYCBPAP |
|  | cg09232555 | 4.23E-03 | -2.18E-01 |  |
|  | cg17440241 | 4.29E-03 | -2.46E-01 | WASF3; WASF3 |
|  | cg00056433 | 4.66E-03 | -2.56E-01 |  |
|  | cg06882249 | 4.90E-03 | -4.05E-01 |  |
|  | cg13824270 | 4.98E-03 | -3.16E-01 | PRPF4B |
|  | cg02680427 | 5.04E-03 | -2.38E-01 |  |
|  | cg05350293 | 5.08E-03 | -2.11E-01 |  |
|  | cg03441844 | 5.17E-03 | -2.16E-01 |  |
|  | cg04785931 | 5.55E-03 | -2.58E-01 |  |
|  | cg19084031 | 5.86E-03 | -2.90E-01 |  |
|  | cg13345255 | 6.05E-03 | -2.03E-01 | SIM1 |
|  | cg05457730 | 6.33E-03 | -2.04E-01 |  |
|  | cg24299306 | 6.97E-03 | -2.01E-01 |  |
|  | cg06747456 | 7.03E-03 | -2.45E-01 |  |
|  | cg00440468 | 7.20E-03 | -3.72E-01 |  |
|  | cg03660901 | 7.50E-03 | -2.41E-01 |  |
|  | cg22857963 | 7.58E-03 | -2.02E-01 | GLI4 |
|  | cg02238229 | 7.94E-03 | -2.21E-01 |  |
|  | cg06777732 | 7.97E-03 | -2.70E-01 |  |
|  | cg20111217 | 8.04E-03 | -2.26E-01 | MYCBPAP |
|  | cg19642077 | 8.29E-03 | -2.17E-01 | SCRIB; SCRIB; SCRIB; SCRIB |
|  | cg24310133 | 8.91E-03 | -2.07E-01 | SLC37A3; SLC37A3 |
|  | cg03894796 | 9.79E-03 | -2.05E-01 |  |
|  | cg08479752 | 1.05E-02 | -2.50E-01 | VSTM1 |
|  | cg15490784 | 1.07E-02 | -2.15E-01 | KLF15 |
|  | cg08124030 | 1.07E-02 | -2.05E-01 | TM4SF1 |
|  | cg25345365 | 1.12E-02 | -2.55E-01 | ZBTB16; ZBTB16 |
|  | cg08164151 | 1.20E-02 | -2.79E-01 |  |
|  | cg11388673 | 1.20E-02 | -2.12E-01 |  |
|  | cg26113488 | 1.29E-02 | -2.04E-01 |  |
|  | cg04998153 | 1.30E-02 | -2.02E-01 | LINC01307 |
|  | cg10071929 | 1.38E-02 | -2.68E-01 | CIZ1; CIZ1; CIZ1; CIZ1; CIZ1 |
|  | cg03540175 | 1.39E-02 | -2.04E-01 | CCDC36; CCDC36; CCDC36 |
|  | cg26220594 | 1.40E-02 | -3.31E-01 |  |
|  | cg09972436 | 1.44E-02 | -4.32E-01 | LCE3C |
|  | cg05740244 | 1.48E-02 | -2.38E-01 | LDHC; LDHC |
|  | cg25960393 | 1.51E-02 | -3.06E-01 |  |
|  | cg16994041 | 1.71E-02 | -2.31E-01 | PRKAG2; PRKAG2 |
|  | cg07318076 | 1.73E-02 | -2.74E-01 | C10orf67 |
|  | cg01996567 | 1.98E-02 | -2.49E-01 | SSPO |
|  | cg12313868 | 2.07E-02 | -2.38E-01 |  |
|  | cg06274109 | 2.09E-02 | -2.24E-01 |  |
|  | cg21367232 | 2.15E-02 | -2.06E-01 | SCAMP2 |
|  | cg17200084 | 2.35E-02 | -2.01E-01 | CAGE1; CAGE1; CAGE1 |
|  | cg10769937 | 2.48E-02 | -2.73E-01 |  |
|  | cg23586821 | 2.49E-02 | -2.37E-01 |  |
|  | cg17586533 | 2.50E-02 | -2.45E-01 | TMEM70; TMEM70; TMEM70 |
|  | cg27466466 | 2.57E-02 | -2.20E-01 |  |
|  | cg16784507 | 2.64E-02 | -2.21E-01 |  |
|  | cg00496389 | 2.70E-02 | -2.70E-01 |  |
|  | cg09399716 | 2.83E-02 | -2.32E-01 |  |
|  | cg00463901 | 3.00E-02 | -2.36E-01 |  |
|  | cg09144455 | 3.03E-02 | -2.04E-01 |  |
|  | cg26173986 | 3.04E-02 | -2.68E-01 | VAMP2 |
|  | cg14239983 | 3.28E-02 | -2.37E-01 | SORL1 |
|  | cg27002237 | 3.65E-02 | -2.14E-01 | TP53AIP1; TP53AIP1; TP53AIP1 |
|  | cg25069157 | 3.67E-02 | -3.67E-01 | TMEM63B |
|  | cg17737388 | 3.79E-02 | -3.58E-01 | C1orf52; C1orf52 |
|  | cg08322244 | 4.07E-02 | -2.20E-01 | VAMP2 |
|  | cg13994730 | 4.72E-02 | -2.17E-01 | LOC100132707; LOC100132707 |
|  | cg20840591 | 4.73E-02 | -2.24E-01 |  |
|  | cg18692070 | 4.73E-02 | -2.02E-01 |  |
|  | cg08977827 | 4.92E-02 | -2.27E-01 |  |
|  | cg13642260 | 4.98E-02 | -2.11E-01 | CIZ1; CIZ1 |
