# Supplementary figures and images for: Genome-wide DNA methylation profiling of CD4+ T lymphocytes identifies differentially methylated loci associated with adult primary refractory immune thrombocytopenia
Source: BMC Med Genomics. 2023 Jun 8;16:124. doi: 10.1186/s12920-023-01557-0 (PMC10251572; doi:10.1186/s12920-023-01557-0)

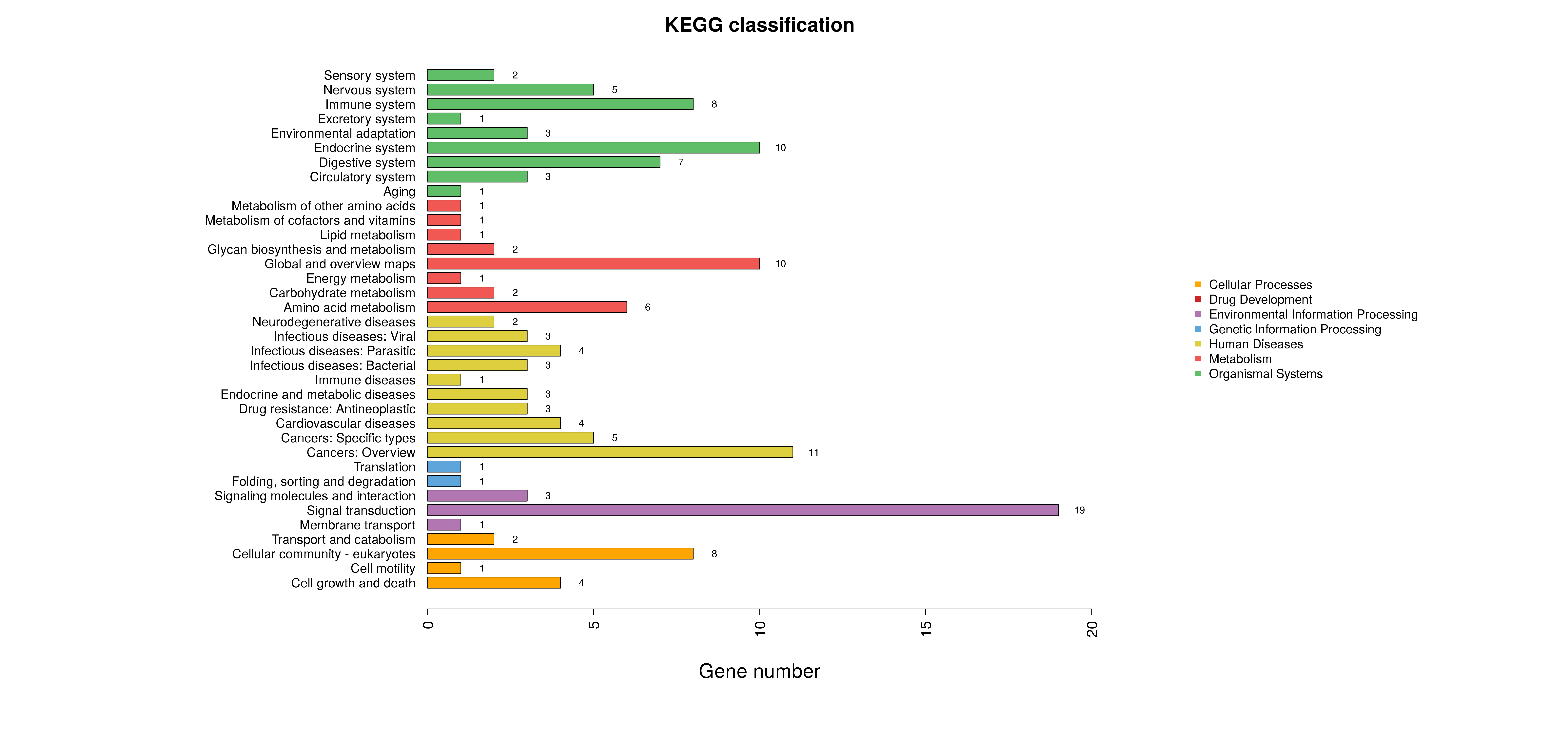

Supplement: Supplementary file 5 — Supplementary Material 5 [file 12920_2023_1557_MOESM5_ESM.tiff]

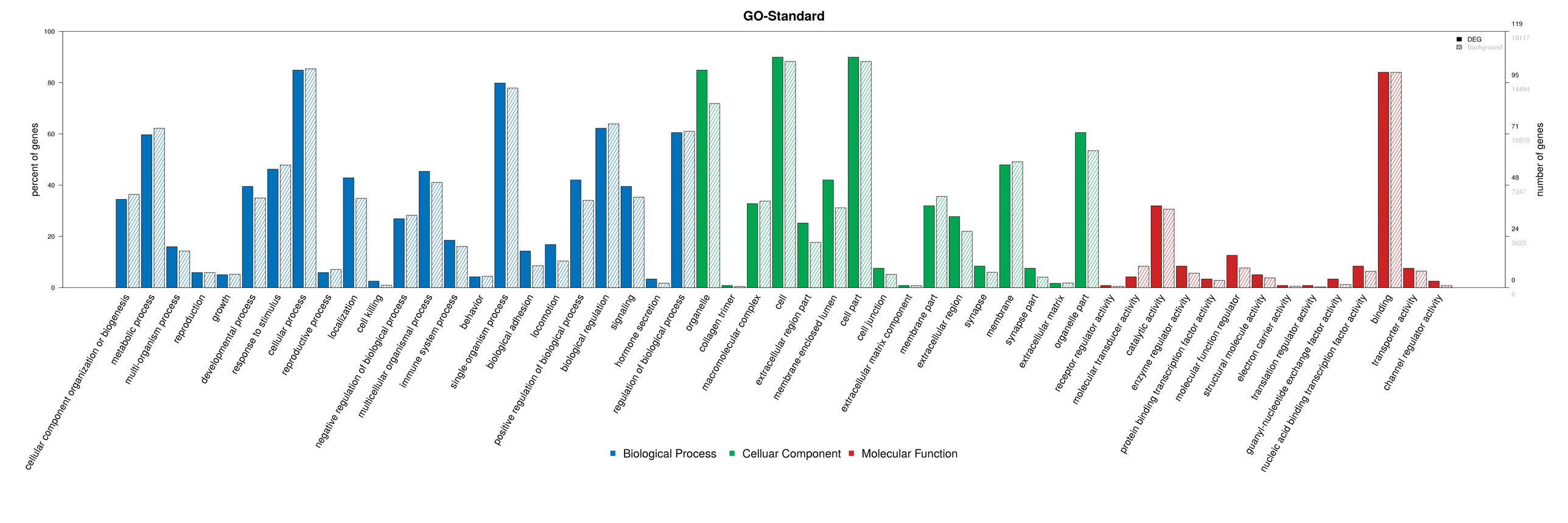

Supplement: Supplementary file 7 — Supplementary Material 7 [file 12920_2023_1557_MOESM7_ESM.tiff]

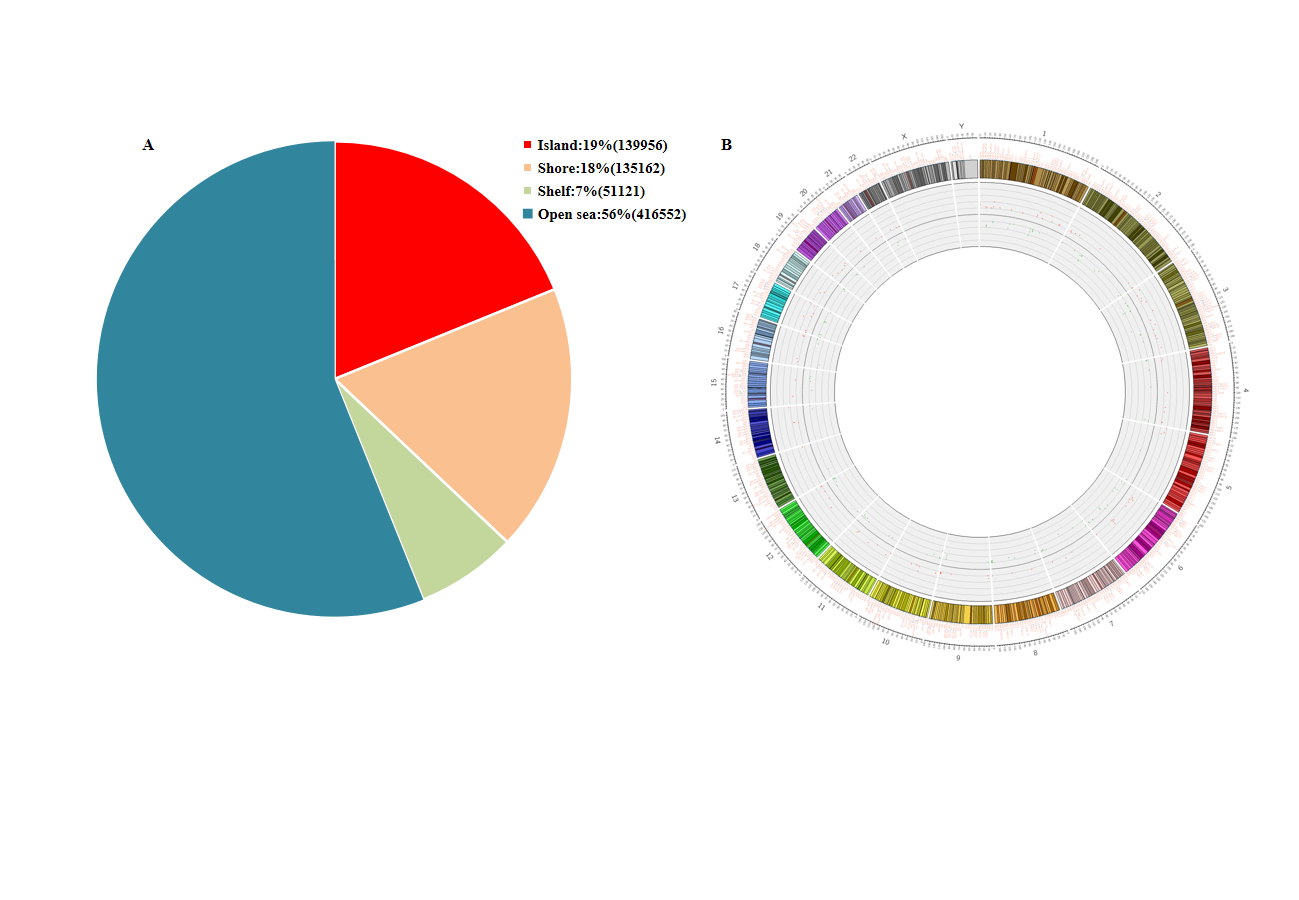

Supplement: Supplementary file 9 — Supplementary Material 9 [file 12920_2023_1557_MOESM9_ESM.tif]

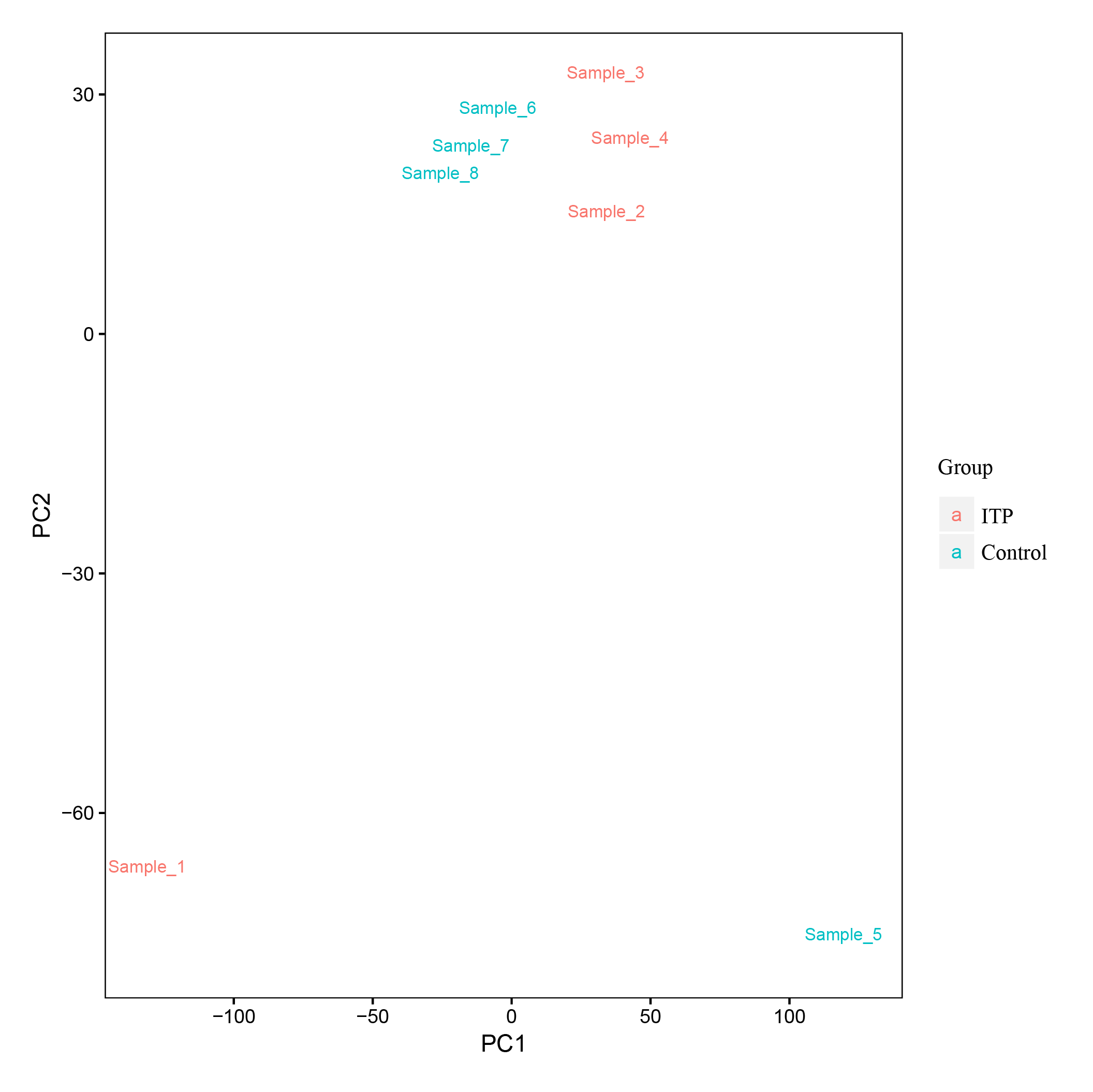

Supplement: Supplementary file 10 — Supplementary Material 10 [file 12920_2023_1557_MOESM10_ESM.tif]
