# Supplementary material for: Genome-wide DNA methylation profiling of CD4+ T lymphocytes identifies differentially methylated loci associated with adult primary refractory immune thrombocytopenia
Source: BMC Med Genomics. 2023 Jun 8;16:124. doi: 10.1186/s12920-023-01557-0 (PMC10251572; doi:10.1186/s12920-023-01557-0)
Supplement: Supplementary file 11 — Supplementary Material 11 [file 12920_2023_1557_MOESM11_ESM.docx]

**Supplementary information**

The primer sequences of each gene are shown in Additional File 1. The list of differential methylation loci will be submitted to Additional File 2. The full DMP data are available as Additional File 3. The complete data of DMRs are provided as Additional File 4. The similarity between the two groups of samples is shown in Supplementary Figure 1 (MDSplot). All detected CPG sites and differential methylation loci distribution on the genome are provided in Supplementary Figure 2A and B. Enrichment analyses of these differentially methylated loci are provided in Additional File 5, Additional File 6, and Supplementary Figure 3 and as well as Supplementary Figure 4.
